# Development and characterization of a spring hexaploid wheat line with no functional *VRN2* genes

**DOI:** 10.1007/s00122-016-2713-3

**Published:** 2016-04-25

**Authors:** Nestor Kippes, Andrew Chen, Xiaoqin Zhang, Adam J. Lukaszewski, Jorge Dubcovsky

**Affiliations:** 1 Department of Plant Sciences, University of California, Davis, CA 95616 USA; 2 Department of Botany and Plant Sciences, University of California, Riverside, CA 92521 USA; 3 Howard Hughes Medical Institute and Gordon and Betty Moor Foundation Investigator, Davis, USA

## Abstract

*****Key message***:**

**The combination of three non-functional alleles of the flowering repressor** ***VRN2*** **results in a spring growth habit in wheat.**

**Abstract:**

In temperate cereals with a winter growth habit, a prolonged exposure to low temperatures (vernalization) accelerates flowering. Before vernalization, the *VRN2* locus plays a central role in maintaining flowering repression. Non-functional *VRN2* alleles result in spring growth habit and are frequent in diploid wheat and barley. However, in hexaploid wheat, the effect of these non-functional *VRN2* alleles is masked by gene redundancy. In this study, we developed a triple *VRN2* mutant (synthetic *vrn2*-null) in hexaploid wheat by combining the non-functional *VRN*-*A2* allele present in most polyploid wheats with a *VRN*-*B2* deletion from tetraploid wheat, and a non-functional *VRN*-*D2* allele from *Aegilops tauschii (Ae. tauschii)* (the donor of hexaploid wheat D genome). Non-vernalized *vrn2*-null plants flowered 118 days (*P* < 2.8E−07) earlier than the winter control, and showed a limited vernalization response. The functional *VRN*-*B2* allele is expressed at higher levels than the functional *VRN*-*D2* allele and showed a stronger repressive effect under partial vernalization (4 °C for 4 weeks), and also in non-vernalized plants carrying only a functional *VRN*-*B2* or *VRN*-*D2* in heterozygous state. These results suggest that different combinations of *VRN*-*B2* and *VRN*-*D2* alleles can be a used to modulate the vernalization response in regions with mild winters. Spring *vrn2*-null mutants have been selected repeatedly in diploid wheat and barley, suggesting that they may have an adaptative value and that may be useful in hexaploid wheat. Spring wheat breeders can use these new alleles to improve wheat adaptation to different or changing environments.

**Electronic supplementary material:**

The online version of this article (doi:10.1007/s00122-016-2713-3) contains supplementary material, which is available to authorized users.

## Introduction

Wheat is an important source of calories and protein worldwide, and within the cereals is the most widely cultivated. Wheat’s adaptability to different environments is favored by a large allelic diversity at the loci that regulate reproductive development in response to seasonal signals, including photoperiod and temperature. Wheat flowering is accelerated under long day photoperiods by the *PHOTOPERIOD1* (*PPD1*) gene (Beales et al. 2007; Wilhelm et al. 2009; Diaz et al. 2012). However, even under inductive photoperiods, wheat varieties with a winter growth habit (winter wheats) require a prolonged exposure to cold temperatures (vernalization) to become competent to flower. Winter wheats are planted in the fall, and the vernalization requirement protects the fragile flowering meristems from being exposed to freezing temperatures during the winter. By contrast, spring wheats are usually planted in the spring, and they have either no vernalization requirement or a very limited response to vernalization.

The vernalization requirement in the temperate cereals is controlled by four *loci* (*VRN1*, *VRN2*, *VRN3* and *VRN*-*D4*) and natural variation at these loci has been associated with different levels of vernalization requirement both in wheat and barley (Yan et al. 2003, 2004a, b, 2006 ; Fu et al. 2005; Kippes et al. 2014, 2015). Both *VRN1* and its paralog *VRN-D4* encode a protein with high similarity to Arabidopsis meristem identity protein APETALA1 (AP1) (Yan et al. 2003; Kippes et al. 2015). Polymorphisms in regulatory regions of the promoter or first intron of this MADS-box gene are associated with loss or reduction of the vernalization requirement (Yan et al. 2003, 2004b; Fu et al. 2005; Hemming et al. 2009; Kippes et al. 2015).

In both wheat and Arabidopsis, the *VRN1/AP1* gene is directly up-regulated by the *FLOWERING LOCUS T* gene, which is designated as *TaFT1* or *VRN3* in hexaploid wheat (Yan et al. 2006). The protein encoded by this gene has been shown to travel through the phloem carrying the photoperiodic signal from the leaves to the shoot apical meristem (Corbesier et al. 2007; Tamaki et al. 2007). Once in the meristem, FT forms a protein complex with FD and 14-3-3 proteins that binds to the promoter of *AP1* homologs *OsMADS15* in rice and *VRN1* in wheat, resulting in their transcriptional activation and the initiation of flowering (Taoka et al. 2011; Li et al. 2015). Natural *TaFT1* alleles with high levels of *TaFT1* expression overcome the vernalization requirement and are associated with spring grow habit (Yan et al. 2006). During the fall, *TaFT1* transcript levels are repressed by the flowering repressor *VRN2* (Yan et al. 2004a). However, the upregulation of *VRN1* during the winter prevents the upregulation of *VRN2* in the spring (Chen and Dubcovsky 2012). In the absence of *VRN2*, the increase in the length of the days during the spring results in the induction of *TaFT1*. *TaFT1*, *VRN1* and *VRN2* are part of a positive regulatory feedback loop that once induced promotes an irreversibly transition from the vegetative to the reproductive stage.

The *VRN2* locus includes two tandemly repeated genes named *ZCCT1* and *ZCCT2* that encode proteins carrying a putative zinc finger and a CCT domain (Yan et al. 2004a). The CCT domain is a 43-amino acid region, first described in the Arabidopsis proteins CONSTANS (CO), CONSTANS-like (COL) and TIMING OF CAB1 (TOC1) (Putterill et al. 1995; Strayer et al. 2000; Robson et al. 2001), and is present in multiple regulatory proteins associated with light signaling, circadian rhythms and photoperiodic flowering (Wenkel et al. 2006). Deletions or mutations involving positively charged amino acids at the CCT domain are associated with recessive *ZCCT1* and *ZCCT2* alleles for spring growth habit in both diploid and tetraploid wheat (Yan et al. 2004a; Dubcovsky et al. 2005; Distelfeld et al. 2009).

The *ZCCT* genes act as long day repressors of flowering and their expression is down-regulated by vernalization and short days (Yan et al. 2004a; Trevaskis et al. 2006; Dubcovsky et al. 2006; Chen and Dubcovsky 2012). The *GRAIN NUMBER, PLANT HEIGHT AND HEADING DATE 7* (*GHD7*) gene in rice and sorghum (*SbGHD7, Ma6*) is the closest homolog to *VRN2* and also works as long day flowering repressor (Xue et al. 2008; Murphy et al. 2014).

A *VRN2* homolog is present in *Brachypodium*, but it is not down-regulated by cold; indeed, a survey of several grasses demonstrated that the down-regulation of *VRN2* by cold is unique to core *Pooideae* such as wheat and barley (Woods et al. 2016).

The VRN2 protein interacts in vitro with CO2 (CONSTANTS2) and with members of the NUCLEAR FACTOR-Y (NF-Y) transcription factor family, but the role of these interactions in the regulation of wheat flowering time are still not clear (Li et al. 2011).

Natural variation in *VRN2* has been detected frequently in diploid wheat (*T. monococcum* L. Yan et al. 2004a) and barley (von Zitzewitz et al. 2005) but not in hexaploid wheat, where gene redundancy likely masks the effect of recessive *vrn2* mutations. A tetraploid wheat line with no functional copies of *VRN2* was previously developed (Distelfeld et al. 2009) by introgressing both a non-functional *vrn*-*A* ^*m*^ *2* allele from *T. monococcum* accession DV92 (Yan et al. 2004a) and a *vrn*-*B2* allele with a large deletion including *ZCCT*-*B1* and the two *ZCCT*-*B2* copies present in this locus (Distelfeld et al. 2009). Using this genetic stock it was demonstrated that most tetraploid wheat accessions carry a non-functional *VRN*-*A2* allele with mutations in critical positively charged amino acids of the CCT domain (Distelfeld et al. 2009). The only exception was *T. turgidum* ssp. *dicoccoides* accession 10-85 collected at Amiad (Israel), which showed no mutations in the CCT domain from *ZCCT*-*A1*.

Since hexaploid wheat is grown in a wider range of environments than tetraploid wheat and represents more than 95 % of the wheat currently grown in the world (Taylor and Koo 2015), a *vrn2*-null genetic stock in hexaploid wheat would be a useful tool to test the adaptative value of *VRN2* loss-of-function mutations in the more diverse environments where hexaploid wheat is grown. In this study, we introgressed a non-functional *vrn*-*D2* of *Aegilops tauschii* in hexaploid wheat, and combined it with *vrn*-*A2* and *vrn*-*B2* non-functional loci to generate a *vrn2*-null genetic stock in hexaploid wheat. Using this genetic stock we demonstrate that simultaneous mutations in all three *VRN2* homologs result in a spring growth habit in hexaploid wheat. Using different combinations of functional and non-functional homoeologs we also show that functional *VRN*-*B2* confers a stronger vernalization requirement than functional *VRN*-*D2*.

## Methods

### Plant materials

The non-functional *vrn*-*D2* allele was obtained from the early flowering diploid *Aegilops tauschii* E1 (Dudnikov 2003). To confirm the presence of the recessive *vrn*-*D2* allele, E1 was crossed with *Ae. tauschii* AS75, a winter accession previously described by Luo et al. (2009). The F_1_ was self-pollinated and an F_2_ segregating population including 71 individuals was generated. Heading data from this population was used to validate the recessive *vrn*-*D2* allele from E1 and to characterize the dominance of the *VRN*-*D2* allele from AS75. The degree of dominance was calculated using the formula: *D* = (2*X*_2_ − *X*_1_ − *X*_3_)/(*X*_1_ − *X*_3_) (Falconer 1964), where *X*_1_, *X*_2_ and *X*_3_ are the heading time values of plants homozygous for the non-functional *vrn*-*D2* allele, the heterozygotes, and plants homozygous for the wild type late flowering *VRN*-*D2* allele, respectively.

The diploid E1 line was then crossed with tetraploid wheat line #3089 (BC_3_F_2_-521 x Kronos, Distelfeld et al. 2009) which carries non-functional alleles for *vrn*-*A2* and *vrn*-*B2*. Chromosome doubling of the triploid hybrid resulted in a synthetic hexaploid with no functional copies of *VRN2*, designated hereafter as “synthetic *vrn2*-null”, which was deposited in the National Small Grain Collection (PI 676269).

To confirm the effect of the non-functional *vrn2* alleles the synthetic *vrn2*-null hexaploid was crossed with Goodstreak (PI 632434, Baenziger et al. 2004). Goodstreak is a hexaploid winter wheat developed by the University of Nebraska that carries alleles for winter growth habit at the three *VRN1* homoeologs and functional copies of *VRN*-*B2* and *VRN*-*D2*. The winter wheat line Triple Dirk C (TDC, Pugsley 1971) and a winter sister line of the synthetic *vrn2*-null carrying functional *VRN*-*B2* and *VRND2* alleles were used to compare the relative transcript levels of these genes by qRT-PCR.

### Growth conditions

Plants were grown in plastic cones (656 ml, 25 cm (9.8 in.) deep × 6.4 cm (2.5 in.) diameter (Steuwe and Sons, USA). Evaluations of plant heading times without vernalization were conducted at UC Davis in a greenhouse with a temperature of 20–25 °C and a photoperiod of 16 h of light (long day). The natural daylight in the greenhouses was supplemented with incandescent lamps at night to extend the photoperiod to 16 h. Vernalization treatments were performed in a growth chamber at constant 4 °C under long day conditions.

For the partial vernalization experiment, plants were grown for one week in the greenhouse, and were then transferred to a 4 °C chamber (16 h of light) for 2, 4 or 6 weeks of vernalization treatment. Plants included in the different treatments were planted at intervals of 2 weeks, so all the plants completed their respective vernalization treatments at the same time. All plants were then transferred together to the same greenhouse (20–25 °C, 16 h of light). Heading time was recorded at the time when half of the spike emerged from the flag leaf.

### Molecular markers

Since the recessive *vrn*-*B2* allele is the result of deletion of all the *ZCCT* genes in this locus, we designed a cleavage amplification polymorphism (CAP) marker for the tightly linked *SNF*-*B2* gene. Primers SNF-B2-3p-F1 and SNFB2-3p-R2 (Table S1) were used to amplify a 1000-bp product. Digestion of the amplified product with restriction enzyme *HpyCH4*IV yielded two fragments (816 and 184 bp) for the synthetic *vrn2*-null and three fragments (626-bp, 188 bp and 184 bp) for hexaploid winter wheat Goodstreak.

For screening of the non-functional *vrn*-*D2* allele, we developed a CAP marker using primers ZCCT-D1-E1-CAPS-F1 and ZCCT-D1-E1-CAPS-R1 (Table S1). These primers amplified an 800 bp product including the first exon of *ZCCT*-*D1*. Digestion of the amplified product with restriction enzyme *Mbo*II yielded two fragments of 650 bp and 150 bp in hexaploid wheat Goodstreak and a single 800 bp product in *Ae. tauschii* E1. Digestion products were separated on 6 % polyacrylamide gels stained with 2 % ethidium bromide. PCR amplifications were performed at 94 °C for 5 min, followed by 40 cycles of 94 °C for 30 s, annealing at 62 °C for 30 s, and extension at 72 °C for 1 min/kb. A final extension step was carried at 72 °C for 7 min.

For screening of the *VRN*-*A1* alleles we used primers developed by Fu et al. (2005) that detect the presence or absence of a large deletion in the first intron of *VRN*-*A1.* These primers were used to differentiate the *VRN*-*A1* allele for spring growth habit present in Kronos (intron deletion) from the *vrn*-*A1* allele for winter growth habit (no intron deletion) present in Goodstreak.

### Identification of mutations in *ZCCT1* and *ZCCT2* of *Aegilopstauschii* E1

Genomic sequences of *ZCCT*-*1A* ^*m*^, *ZCCT*-*2A* ^*m*^ from *Triticum monococcum*, and *ZCCT*-*A1*, *ZCCT*-*B1*, *ZCCT*-*D1*, *ZCCT*-*A2* from *Triticum aestivum* were retrieved from NCBI (http://www.ncbi.nlm.nih.gov/). Additional sequences of *ZCCT*-*B2* and *ZCCT*-*D2* were retrieved from the genomic contigs database from the International Wheat Genome Sequencing Consortium website (IWGSC, http://wheat-urgi.versailles.inra.fr). Complete sequences of the *ZCCT1* and *ZCCT2* homologs including their 5′ and 3′ UTRs were aligned using T-COFFEE (www.tcoffee.org).

All *ZCCT1* and *ZCCT2* homologs have two exons separated by a large intron. The two exons from *ZCCT1* and *ZCCT2* were PCR-amplified separately from *Ae. tauschii* accession E1 using primers and conditions listed in Table S1. Genomic DNA sequences were obtained by Sanger sequencing using an Applied Biosystems 3730XL DNA Analyzer, and were assembled using GAP4 v1.5 (Bonfield et al. 1995).

To confirm different splicing variants detected for *ZCCT*-*D1* in *Ae. tauschii* E1, RNA was extracted using the Spectrum™ Plant Total RNA kit (SIGMA) from leaves of two-week old plants grown without vernalization under long day photoperiod. cDNAs were produced using the High Capacity Reverse Transcription Kit (Applied Biosystems) according to manufacturer’s instructions. Specific primers ZCCT-D1-F, ZCCT-D1-R, ZCCT-D2-F, and ZCCT-D2-R and PCR annealing temperatures are listed in Table S1. Cloning was performed using NEB^®^ PCR Cloning Kit (New England Biolabs, USA). Positive colonies were grown and used for colony PCR and the product confirmed by Sanger sequencing.

### *ZCCT2* transcript levels in hexaploid wheat

Plants were grown for 3 weeks in a PGR15 growth chambers (Conviron) under long day conditions (16 h light/8 h dark) at a temperature of 20 °C during light period and 18 °C during dark. Samples were collected 4 h after the lights were turned on (Zeitgeber Time 4). RNA was extracted from leaves using the Spectrum Plant Total RNA Kit (Sigma-Aldrich). Quantitative PCR was performed using SYBR Green and a 7500 Fast Real-Time PCR system (Applied Biosystems). Primers for ACTIN are described in Distelfeld et al. (2009) and primers for *ZCCT*-*B2* and *ZCCT*-*D2* are described in Table S1. Transcript levels are expressed as linearized fold-*ACTIN* levels calculated by the formula 2^(*ACTIN* CT – *TARGET* CT)^ ± SE of the mean. The resulting number indicates the ratio between the initial number of molecules of the target gene and the number of molecules of *ACTIN*.

## Results

### Validation of the non-functional *vrn*-*D2* allele in *Ae. tauschii* E1

**Fig. 1:**
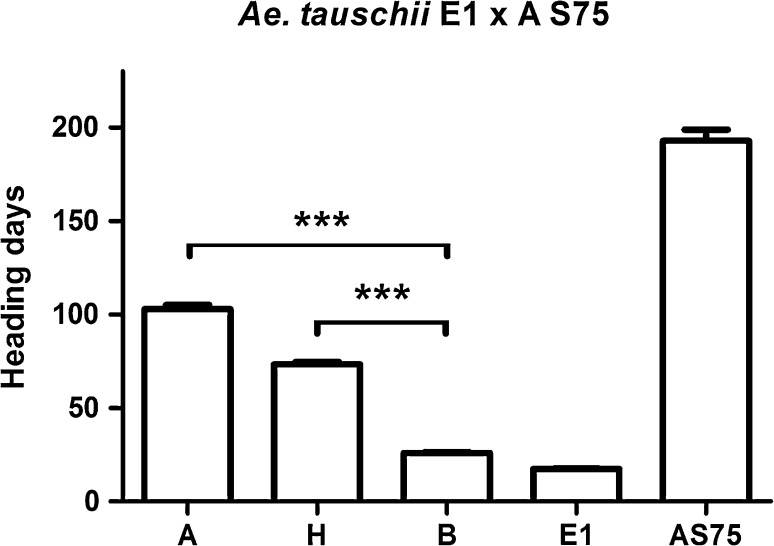
Effect of *VRN2* mutations on flowering time in diploid *Ae. tauschii*. Heading time of 71 F_2_ plants from the cross between *Ae. tauschii* accessions *E1* (early flowering) and *AS75* (late flowering). *A* = AS75 allele, *B* = *Ae. tauschii* E1 allele and *H* = heterozygous. *E1* = *Ae. tauschii* E1 and *AS75* = *Ae. tauschii* AS75 indicate parental controls. Plants were grown under long days in the absence of vernalization. ***P < 0.001

We first confirmed the presence of the non-functional *vrn2* allele in the seeds received for *Ae. tauschii* accession E1. The F_2_ population from a cross between *Ae. tauschii* AS75 and E1 accessions showed the expected 1:2:1 segregation (16:33:22, χ^2^ *P* = 0.51) for *VRN*-*D2*. All the F_2_ plants homozygous for the E1 *VRN*-*D2* allele headed very early (average 26.1 ± 0.5 d), and those homozygous for the AS75 allele headed very late (average 102.8 ± 2.4 d), confirming that the E1 accession has a recessive *vrn*-*D2* allele (Fig. 1).

The heterozygous plants showed an intermediate flowering time (average 73.3 ± 1.3 days), which was slightly closer to the average of the plants homozygous for the AS75 allele than to those homozygous for the E1 allele (Fig. 1). The degree of dominance of the functional *VRN*-*D2* allele for heading time was 23 % in this population. A very similar degree of dominance (26 %) was reported before in a tetraploid population segregating only for the functional *VRN*-*B2* allele (Distelfeld et al. 2009).

### Molecular characterization of *ZCCT*-*D1* and *ZCCT*-*D2* sequences

To better understand the nature of the recessive *vrn*-*D2* allele in E1, we sequenced the *ZCCT*-*D1* and *ZCCT*-*D2* genes from this accession and compared it to the sequence of winter lines AS75 and AL8/78. The primers used in this study are described in supplemental Table S1.

**Fig. 2:**
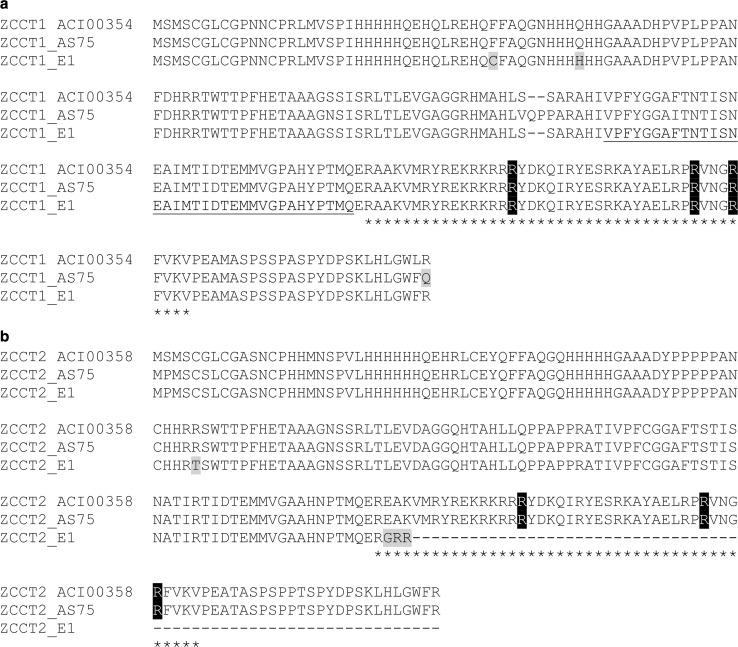
Mutations in the *VRN* *2* locus of *Ae. tauschii* E1. Multiple alignments of predicted coding sequences of *ZCCT1* **a** and *ZCCT2* **b** in *Ae. tauchii* E1 (KM489155 and KM489156), and winter controls AS75 (KM503042 and KM503043) and AL8/78 (ACI00354 and ACI00358). Numbers in parenthesis are GenBank accession numbers for ZCCT1 and ZCCT2, respectively. For the ZCCT1 protein from E1, a 35 amino acid deletion observed in 87.5 % of the cloned and sequenced cDNAs is* underlined* (the other 12.5 % of the clones include an intron region producing a premature stop codon). The CCT domain is indicated with* asterisks* in both genes. SNPs are highlighted in* gray*. Critical Arg residues in the CCT domain are highlighted in* black*

The predicted protein sequence of ZCCT-D1 from E1 (KM489155) showed two amino acid changes (F36C and Q45H) that differentiated E1 from the two winter lines. A two amino acid deletion at positions 101 and 102 was observed in both the early flowering E1 and the late flowering AL8/78, and therefore, is not likely to affect the *VRN2* function (Fig. 2a, Fig. S1). According to the SIFT score (Ng and Henikoff 2001), the amino acid substitution in F36C has a high probability to alter protein structure or function (Fig. 2a, Fig. S1). The deletion at positions 101 and 102 (also found in ZCCT-D1, GenBank accession ACI00354) is absent in the ZCCT-A1 and ZCCT-B1 sequences (Fig. S1).

The genomic *ZCCT*-*D1* sequence of E1 has a 24 bp deletion located 6 bp before the splicing acceptor site at the 3′ end of the single intron that is not observed in the two winter *Ae. tauschii* accessions sequenced in this study (Fig. S2). To test the effect of this intron deletion on splicing, we cloned and sequenced multiple *ZCCT*-*D1* transcripts from *Ae. tauschii* E1 and the winter control AS75. In E1, 21 of the 24 sequenced clones (87.5 %) showed an alternative splicing variant that generated a deletion of 35 amino acids before the CCT domain (region underlined in Fig. 2a). The other three clones (12.5 %) included a 58-bp region of the intron producing a premature stop codon and disrupting the CCT domain. None of the clones obtained from E1 showed a normal and complete *VRN2* transcript, indicating that different acceptor sites were used for all the *ZCCT*-*D1* transcripts. By contrast, the winter control AS75 showed a single splicing product encoding the complete *ZCCT*-*D1* gene.

The *ZCCT*-*D2* genomic sequence showed one amino acid change and a 1 bp deletion that were present only in E1. The 1 bp deletion generates a change in the reading frame that disrupts the CCT domain (Fig. 2b). These changes were confirmed by sequencing 37 cDNA clones of *ZCCT*-*D2* from E1. Taken together, the molecular and the genetic data indicate that E1 carries a non-functional *VRN2* allele.

### Development and characterization of a triple *VRN2* mutant in hexaploid wheat

The E1 accession carrying the non-functional *VRN2* allele was crossed with tetraploid line #3089 carrying non-functional *VRN*-*A2* and *VRN*-*B2* alleles (Distelfeld et al. 2009, Fig. 3). The triploid F_1_ hybrids were treated with colchicine (0.1 % for 7–7:30 h), but they were very susceptible to colchicine and most of them died. Fortunately, the triploid F_1_ hybrids naturally produced some fertile seeds. Cytogenetic characterization of these seeds confirmed the chromosome doubling (2*n* = 42), which likely occurred by meiotic restitution.

**Fig. 3:**
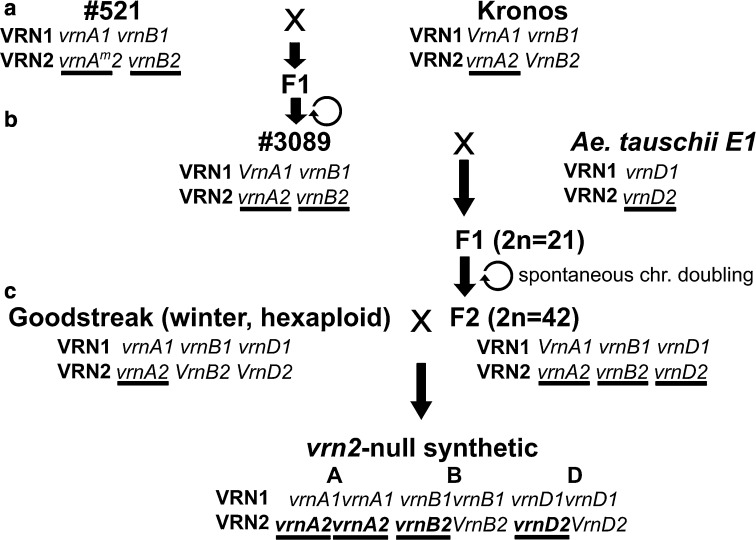
Development of synthetic *vrn2*-null hexaploid wheat. **a** Introgression of *vrn*-*A2* and *vrn*-*B2* in the tetraploid cultivar Kronos. **b** Production of synthetic aneuploid to introgress *vrn*-*D2*, self-pollination to recover chromosome number. **c** Backcross with winter hexaploid wheat to produce a segregating line for *vrn*-*A2*, *vrn*-*B2* and *vrn*-*D2* in a winter background. *X* represents a crossing step, *arrow* represents a progeny. Non-functional *VRN2* alleles are *underlined*

**Fig. 4:**
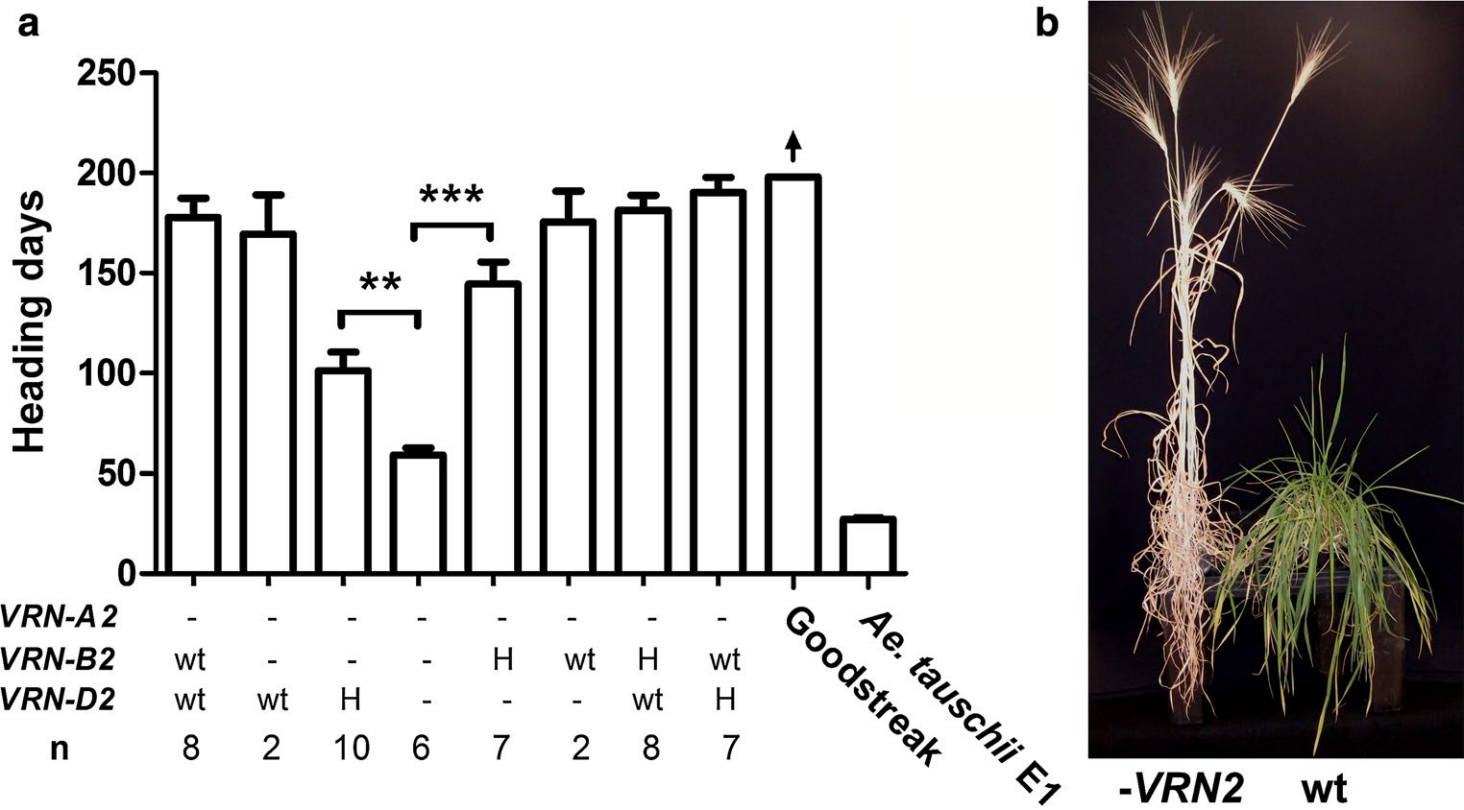
Effect of mutations in *VRN*-*B2* and *VRN*-*D2* on heading time under non vernalizing conditions. **a** Heading times for the triple mutant. *Bars* represent mean and error bars represent the SEM. *Dashes* represent mutant alleles, wt: wild type alleles and H: heterozygous. Lower doses of functional *VRN2* were enough to significantly delay flowering. **P < 0.005 ***P < 0.001. *Arrow* indicates that the experiment was stopped before heading. **b** Maturity differences between the triple *VRN2* mutant (*left*) and the wild type (*right*)

The resulting synthetic hexaploid carried non-functional copies of all three *VRN2* homoeologs (synthetic *vrn2*-null), but it also carried a dominant *Vrn*-*A1* allele for spring growth habit that complicated the analysis. To eliminate the *VRN*-*A1* allele, the *vrn2*-null synthetic was crossed with the winter wheat variety Goodstreak that carries recessive *vrn*-*A1*, *vrn*-*B1*, and *vrn*-*D1* alleles (Fig. 3). Both parental lines carry the non-functional *VRN*-*A2* allele. The F_1_ hybrid was self-pollinated and the segregating population (93 F_2_ plants) was genotyped with markers for *VRN*-*B2*, *VRN*-*D2* and *VRN*-*A1*. Only two plants were triple homozygous *vrn*-*A1*, *vrn*-*B2* and *vrn*-*D2* and both were early flowering. To increase the number of plants segregating for only for *VRN*-*B2* and *VRN*-*D2*, we selected F_2_ plants homozygous for the *vrn*-*A1* allele for winter growth habit and heterozygous for the two *VRN2* genes. In this segregating F_3_ population (69 plants), we identified 6 plants homozygous for the non-functional *vrn*-*A2*, *vrn*-*B2* and *vrn*-*D2* plants. These plants headed more than 100 days earlier than those homozygous for at least one of the functional *VRN2* alleles (*P* < 2.8E–07) in the absence of vernalization (Fig. 4).

### Relative effect of *VRN*-*B2* and *VRN*-*D2* on flowering time and vernalization response

In the absence of vernalization, plants carrying only the functional *VRN*-*B2* or the functional *VRN*-*D2* allele in homozygous state exhibited a winter growth habit, and showed no significant differences in heading time with the control plants carrying both functional *VRN2* genes (Fig. 4). Plants carrying only the functional *VRN*-*D2* allele flowered six days earlier (169 days) than the plants carrying only the functional *VRN*-*B2* allele (175 days), but the differences were not significant. Interestingly, plants carrying only one functional *VRN2* allele in heterozygous stage flowered significantly earlier than plants homozygous for any of the functional *VRN2* alleles (Fig. 4). Plants carrying only the functional *VRN*-*B2* allele in heterozygous state headed 43 d later than plants carrying only the functional *VRN*-*D2* allele in heterozygous state (144.5 d *vs* 101.2 d, *P* = 0.009, Fig. 4). These results suggest that the functional *VRN*-*B2* allele has a stronger effect in repression of flowering under long days than the *VRN*-*D2* allele (Fig. 4).

**Fig. 5:**
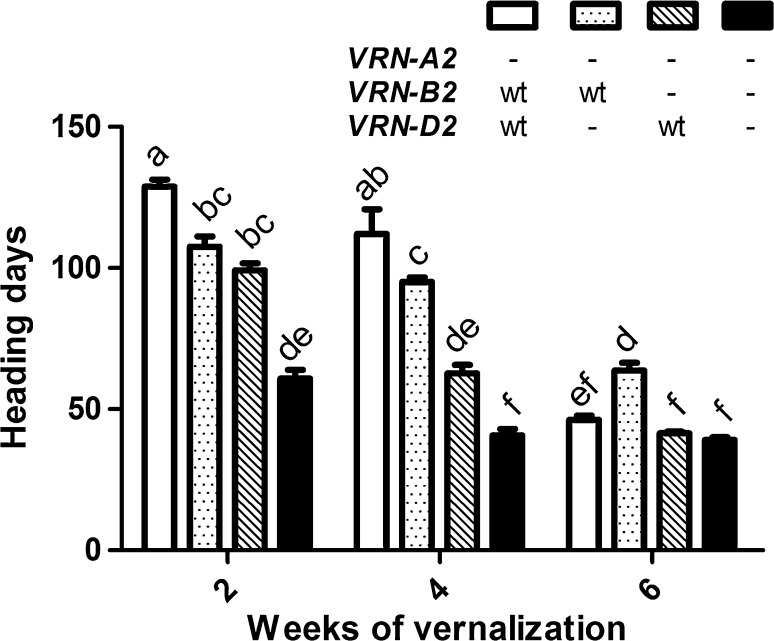
Effects of mutations in *VRN*-*B2* and *VRN*-*D2* on heading time under different vernalization treatments. *Error bars* represent the SE of the mean of 5 biological replications. *Dashes* represent a non-functional allele and wt a wild type allele. For each treatment different letters indicate significant differences (Tukey’s test, *P* < 0.05)

The stronger vernalization requirement of the functional *VRN*-*B2* allele relative to *VRN*-*D2* was also supported by a partial vernalization experiment. After 2 weeks of vernalization, the heading time differences between the functional *VRN*-*B2* and *VRN*-*D2* alleles were still significant, but plants with a single functional *VRN2* allele flowered significantly earlier than the control with both functional alleles (Fig. 5). After 4 weeks of vernalization, plants carrying only the functional *VRN*-*B2* allele headed 32 days later than those carrying the functional *VRN*-*D2* allele (*P* = 1.34E−5).

**Fig. 6:**
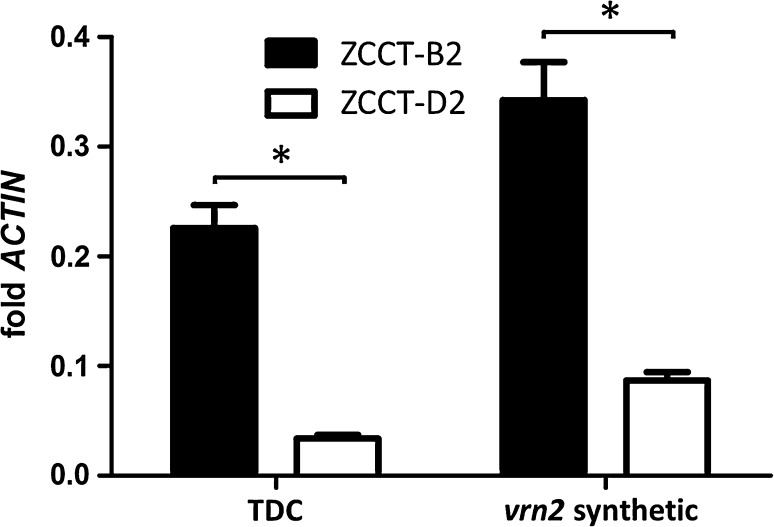
Transcript levels of *ZCCT*-*B2* and *ZCCT*-*D2* in hexaploid wheat. *ZCCT*-*B2* levels are 6 and 4 times higher than *ZCCT*-*D2* in TDC and *vrn2*-synthetic, respectively. Triple Dirk C (TDC) is a winter hexaploid line and *vrn2*-synthetic corresponds to the wild type sister line of the synthetic *vrn2*-null developed in this study. *Bars* represent means of 5 biological and 2 technical replications. Plants were grown under long day conditions (16 h light) at a temperature of 20 °C day/18 °C night for 3 weeks. Samples were collected at ZT 4 (Zeitgeber Time). *P < 0.001

To test if the stronger repressive effect of *VRN*-*B2* relative to *VRN*-*D2* was associated with differences in expression, we compared their transcript levels in two winter wheat lines using qRT-PCR. In a sister line of the synthetic *vrn2*-null carrying functional *VRN*-*B2* and *VRN*-*D2* alleles, the transcript levels of *ZCCT*-*B2* were fourfold higher than those of *ZCCT*-*D2* (*P* < 0.001, Fig. 6). A similar result was observed in the winter line Triple Dirk C, where the transcript levels of *ZCCT*-*B2* were sixfold higher than those of *ZCCT*-*D2* (*P* < 0.001, Fig. 6).

Finally, after 6 weeks of vernalization all genotypes flowered very early although the plants carrying the functional *VRN*-*B2* allele flowered later than the other genotypes (Fig. 5). We do not know the cause of this difference.

As in the previous experiment with non-vernalized plants (Fig. 4), the synthetic *vrn2*-null showed the earliest heading time among the four genotypes for the plants exposed to 2 and 4 weeks of vernalization (Fig. 5). Interestingly, the synthetic *vrn2*-null showed a residual response to vernalization. When these plants were vernalized for 4 weeks, they flowered 20 days earlier (*P* = 0.001) than when they were vernalized for 2 weeks (Fig. 5). No significant differences in heading time were detected between synthetic *vrn2*-null plants vernalized for 4 or 6 weeks (*P* = 0.529, Fig. 5), or between those that were vernalized for 2 weeks (60.8 days) relative to non-vernalized plants (58.0 days, *P* = 0.433).

## Discussion

### *VRN2* natural variation in polyploid wheat

In hexaploid wheat, most of the natural variation in growth habit is associated with dominant mutations in regulatory regions of the three *VRN1* homoeologs (Yan et al. 2004b; Fu et al. 2005; Dubcovsky et al. 2006; Zhang et al. 2008; Chu et al.^2011^; Zhang et al. 2012; Muterko et al. 2015, 2016), and less frequently with dominant mutations in *VRN3* (=*TaFT1*, Yan et al. 2006; Zhang et al. 2008) or *VRN*-*D4* (a *VRN1* paralogue; Kippes et al. 2015). However, no natural variation in growth habit has been associated so far with variation at any of the three *VRN2* homoeologs in hexaploid wheat.

The limited effect of single *VRN2* mutations on heading time in non-vernalized or fully vernalized hexaploid plants may explain why no *vrn2*-null spring varieties have been selected so far in polyploid wheat. By contrast, *vrn2*-null spring accessions are frequent in both *T. monococcum* (Yan et al. 2004b) and barley (von Zitzewitz et al. 2005), supporting the hypothesis that functional redundancy among *VRN2* homoeologs in the polyploid wheat species may have masked the effect of recessive mutations on heading time from natural or human selection.

Although no *vrn2*-null spring accessions of hexaploid wheat have been reported so far, molecular and genetic studies have revealed allelic variation in *ZCCT1* and *ZCCT2* at each of the three *VRN2* homoeologs. Since non-functional mutations in both tightly linked *ZCCT1* and *ZCCT2* genes are required to generate a recessive *VRN2* locus, it is important to describe the allelic variation in both genes within each locus. The initial studies in *T. monococcum* identified *ZCCT1* as the functional gene in the *VRN*-*A* ^*m*^ *2* locus (Yan et al. 2004a), but a subsequent study in tetraploid wheat showed that *ZCCT2* was the functional gene in the *VRN*-*B2* locus (Distelfeld et al. 2009).

Most of the *VRN*-*A2* loci show non-functional mutations in the CCT domains of both *ZCCT*-*A1* and *ZCCT*-*A2* in tetraploid wheat. Only one accession of *T. turgidum* subsp. *dicoccoides* showed a wild type CCT domain for ZCCT-A1 in a collection of 78 tetraploid wheats including both wild and domesticated accessions (Distelfeld et al. 2009). For ZCCT-A2, all 78 accessions have a C16R mutation in the CCT domain. This mutation was also detected in 15 accessions of *T. urartu* and in two accessions of *T. monococcum*, suggesting that this mutation was already fixed (or frequent) before the divergence of these two closely related diploid species (Distelfeld et al. 2009). In tetraploid wheat combination of CCT mutations C39R in ZCCT-A1 and C16R in ZCCT-A2 result in a recessive *vrn*-*A2* allele (Distelfeld et al. 2009). The same mutations are also present in the early flowering synthetic *vrn2*-null hexaploid generated in this study, confirming that this *vrn*-*A2* allele is also non-functional in hexaploid wheat. Zhu et al. (2011) identified six accessions of hexaploid wheat where they were unable to amplify the promoter of *ZCCT*-*A1*, suggesting that these varieties may carry non-functional *vrn*-*A2* alleles. However, *ZCCT*-*A2* was not characterized in the previous study.

The *VRN*-*B2* locus sequenced in tetraploid wheat carries a non-functional ZCCT-B1 protein (C39R mutation in 37 sequenced *T. turgidum* subsp. *durum*), and two recently duplicated copies of *ZCCT*-*B2* (wild type CCT domain). The presence of these two *ZCCT*-*B2* copies is sufficient to confer a strong vernalization requirement (Distelfeld et al. 2009). A complete deletion of all three *ZCCT* genes present in *VRN*-*B2* was identified in one accession of *T. turgidum* subsp. *dicoccon* collected in Turkey (PI470739), and was associated with early flowering in a tetraploid wheat segregating population (Distelfeld et al. 2009). In this study, we confirmed that the *VRN*-*B2* deletion is associated with early flowering and spring growth habit in the synthetic *vrn2*-null hexaploid. A similar deletion in *VRN*-*B2* was recently reported in the hexaploid wheat variety Jagger, but no significant differences in heading time were detected for this locus in a segregating population tested in the field in Oklahoma (USA) under natural vernalization conditions (Tan and Yan 2015).

More limited allelic variation has been described for the *VRN*-*D2* locus. *Ae. tauschii* accessions AS75 has a winter growth habit and the predicted ZCCT-D1 and ZCCT-D2 proteins show no mutations in the conserved amino acids of the CCT domains (Distelfeld et al. 2009; Tan and Yan 2015). Zhu et al. (2011) identified one accession from Macedonia (CItr 17383) with a deletion in the *ZCCT*-*D1* promoter, but this accession was later reclassified in GRIN as *T. turgidum* (2*n* = 28), explaining the absence of the D genome locus. The limited variability in the D genome of hexaploid wheat observed in this locus prompted us to look for variation in *Ae. tauschii* to identify a non-functional *VRN*-*D2* allele.

### *Ae. tauschii* E1 carries a recessive *vrn*-*D2* allele

The early flowering in *Aegilops tauschii* accession E1 was previously reported to be linked to isozyme ACO2 locus on chromosome arm 4DL (recombination fraction 0.32), suggesting that it may correspond to the *VRN*-*D2* locus (Dudnikov 2003). The segregating population generated in this study from the cross between *Ae. tauschii* spring accession E1 and winter accession AS75 confirmed that the differences in heading time were completely linked to polymorphisms in *VRN*-*D2*, suggesting that E1 carries a recessive *vrn*-*D2* allele.

The previous hypothesis was also supported by the presence of disruptive mutations in both *ZCCT*-*D1* and *ZCCT*-*D2* in E1 but not in the two *Ae. tauschii* winter accessions used for comparison.

For *ZCCT*-*D1*, two amino acid changes close to the predicted zinc finger were observed only in E1. One of these changes resulted in an additional cysteine in E1 (F36C), which has the potential to disrupt the zinc finger. Although the previous amino acid changes may affect the function of ZCCT1, the most drastic change in this gene was the presence of altered splice forms probably associated with a 24 bp deletion in the intron. Although this deletion did not include the AG splicing site, it is located only six bp from the splicing acceptor. The deleted region includes a polypyrimidine tract that is known to be important for correct splicing and it also alters the distance between the acceptor site and the putative branch point, which is also important for the selection of the correct splicing site (Black 2003). The analysis of the *ZCCT*-*D1* transcripts confirmed that none of them use the canonical acceptor site. The alternative splice sites were associated either with a 35 amino acid deletion before the CCT domain or with the translation of part of the intron encoding a premature stop codon. For *ZCCT*-*D2*, we detected a frame shift mutation that was present only in E1, and is predicted to result in a protein lacking almost the complete CCT domain (Fig. 2b).

In summary, both *ZCCT*-*D1* and *ZCCT*-*D2* showed disruptive mutations in E1 that are expected to greatly reduce or eliminate *VRN*-*D2* function. This hypothesis was confirmed by the early flowering of the diploid plants carrying the E1 *vrn*-*D2* allele in the diploid segregating population, and also by the early flowering of the synthetic *vrn2*-null synthetic hexaploid including this allele. These results also confirmed that the protein region adjacent to the CCT domain is necessary for proper protein function, and that the intron region adjacent to the acceptor splicing site is critical for correct splicing of the *ZCCT1* transcripts.

### Recessive alleles at the three *VRN2* homologs results in a spring growth habit

With the identification of the recessive *vrn*-*D2*-null allele it was possible to develop a triple *vrn2*-null hexaploid wheat. This *vrn2*-null hexaploid line was crossed with the winter hexaploid wheat Goodstreak to study the effect of the different *vrn*-*2* alleles in the absence of spring alleles in the *VRN1* homoeologs. This is important because dominant *VRN1* alleles for early flowering are epistatic to *VRN2* and can mask their effect (Tranquilli and Dubcovsky 2000; Chen and Dubcovsky 2012).

In the absence of dominant *VRN1* alleles for spring growth habit, non-vernalized plants carrying non-functional *vrn2*-null alleles took roughly 60 days from sowing to heading, whereas plant carrying either the functional *VRN*-*B2* or *VRN*-*D2* alleles (or both) headed more than 100 days later then the *vrn2*-null plants (Fig. 5). These large differences in heading time disappeared when plants were vernalized for 6 weeks (Fig. 5), confirming that the late flowering was associated to a strong vernalization requirement.

Interestingly, synthetic *vrn2*-null hexaploid plants showed a residual response to the vernalization treatment. When these plants were vernalized for 4 or 6 weeks they flowered on average 20 days earlier than when they were vernalized for 2 weeks or were not vernalized at all. Although we cannot completely rule out the possibility that this residual vernalization response is caused by some functionality of the mutant VRN-A2 or VRN-D2 proteins (hypomorphic mutations), the presence of similar effects in other species supports an alternative explanation.

Vernalization of barley plants in which all the *ZCCT* genes have been deleted (*vrn2*-null) still accelerated heading time by 2 weeks (Hemming et al. 2008). In the same study, *VRN1* expression was induced by vernalization in the presence and absence of *VRN2*, suggesting that this gene may contribute to the residual vernalization response of the *vrn2*-null plants. Although we do not disregard a possible contribution of *VRN1*, it was recently demonstrated that tetraploid wheat plants, carrying non-functional copies of both *VRN1* and *VRN2* genes (henceforth, *vrn1*-*vrn2*-null), still flower on average 23 days earlier when they are vernalized than when they are not (Chen and Dubcovsky 2012). Since the *vrn1*-*vrn2*-null plants lack any functional *VRN1* and *VRN2* genes, these results indicate that wheat has additional genes that are able to accelerate flowering in response to vernalization.

### *ZCCT* homoeologs exhibit differential responses under partial vernalization and when present in different dosages

The stronger effect of the functional *VRN*-*B2* allele relative to the functional *VRN*-*D2* allele was evident in the non-vernalized plants carrying only one functional *VRN2* allele in heterozygous state (Fig. 4). Plants carrying only one functional copy of *VRN*-*B2* flowered in average 43 days later than those with only one functional copy of *VRN*-*D2* (Fig. 4, *P* < 0.009). The stronger effect of the functional *VRN*-*B2* allele relative to the *VRN*-*D2* allele was also evident in the partial vernalization response experiments (Fig. 5).

The stronger effect of the *VRN*-*B2* locus on heading time was correlated with four- to sixfold higher transcript levels of *ZCCT*-*B2* relative to those of *ZCCT*-*D2* (Fig. 6). Although these differences in expression are likely sufficient to explain the different effects of these alleles on heading time, we cannot rule out the possibility of additional differences in the strength of the encoded ZCCT-B2 and ZCCT-D2 proteins or the presence of residual activity of the protein encoded by the *vrn*-*D2* recessive allele.

A previous study in tetraploid wheat showed that the *ZCCT2* genes are expressed at 3- to 10-fold higher levels than the *ZCCT1* genes (Distelfeld et al. 2009). This may contribute to the stronger effect of the *VRN*-*B2* allele, which includes two recently duplicated copies of the wild type *ZCCT*-*B2* gene (Distelfeld et al. 2009).

Although plants carrying only a single copy of the functional *VRN*-*B2* allele in heterozygous state flowered more than 40 days later than those carrying only a single copy of the functional *VRN*-*D2* allele in heterozygous state, the differences were no longer significant when these functional alleles were in the homozygous state. No further delays in heading time were observed when both the *VRN*-*B2* and *VRN*-*D2* functional alleles were combined. Taken together, these results suggest that the repressive effect of *VRN2* on heading time may be saturated beyond two functional copies of any of the active alleles. The small phenotypic effect of the non-functional mutations in one of the *VRN2* homoeologues limits the effects of natural or human selection on these alleles. This may explain why no natural *vrn2*-null mutants have been identified so far.

### Conclusions and potential practical applications

Both the single and double *VRN2* mutants generated in this study may have practical applications. The reduced vernalization requirement associated with the presence of single *VRN*-*B2* and *VRN*-*D2* mutants under partial vernalization suggests that these mutants may be useful to modulate the vernalization response in regions with mild winters (e.g., Mediterranean regions) or as global warming reduces the severity of winters in other locations.

It would be also important to study the effect of the absence of all functional *VRN2* alleles (*vrn2*-null) on the duration of the different reproductive phases, and the effect of these changes on hexaploid wheat adaptation to different environments. We speculate that this new class of spring wheat varieties may have practical applications in some environments based on the relatively high frequency of *vrn2*-null spring accession in diploid wheat and barley species. The non-functional *vrn2*-null allele was detected in 80 % of the accessions in a collection of 65 cultivated *T. monococcum* ssp. *monococcum* accessions (Yan et al. 2004a) and is also frequent in barley. Barley varieties carrying the *vrn2*-null allele have been designated as “facultative winters” and have been recommended for some of the barley growing regions (Karsai et al. 2005; Szucs et al. 2007).

Seeds of the synthetic *vrn2*-null hexaploid wheat with no functional homoeologues of *VRN2* have been deposited in the National Small Grain Collection (PI 676269). Seeds from this genetic stock are publicly available and can be used as a source of non-functional *VRN*-*B2* and *VRN*-*D2* alleles in common wheat breeding programs. These new *VRN2* alleles provide common wheat breeders new tools to improve wheat adaptation to new or changing environments.

#### Author contribution statement

CA sequenced VRN2 in E1 and AS75 and performed E1xAS75 progeny test, LA and ZX produced the synthetic hexaploid wheat, KN sequenced coding regions of *ZCCT1* and *ZCCT2* in E1, cloned and sequenced *ZCCT1* and *ZCCT2* amplicons from E1 and controls, carried out the ZCCT2 qRT-PCR, performed the segregation analysis tests with and without vernalization, made the multiple sequence alignments analysis and produced the figures. KN and DJ analyzed data and wrote the manuscript.

## Electronic supplementary material

Below is the link to the electronic supplementary material.

Supplementary material 1 (DOCX 1979 kb)
