# Supplementary material for: Development and characterization of a spring hexaploid wheat line with no functional VRN2 genes
Source: Theor Appl Genet. 2016 Apr 25;129(7):1417–28. doi: 10.1007/s00122-016-2713-3 (PMC4909811; doi:10.1007/s00122-016-2713-3)
Supplement: Supplementary file 1 — Supplementary material 1 (DOCX 1979 kb) [file 122_2016_2713_MOESM1_ESM.docx]

Supplementary Materials for

**Development and characterization of a spring hexaploid wheat line with no functional *VRN2* genes**

Nestor Kippes^1^ · Andrew Chen^1^ · Xiaoqin Zhang^1^ · Adam J Lukaszewski^2^ · Jorge Dubcovsky^1, 3^

* correspondence to: [jdubcovsky@ucdavis.edu](mailto:jdubcovsky@ucdavis.edu)

**This file includes:**

Figures S1, S2 and Table S1

**
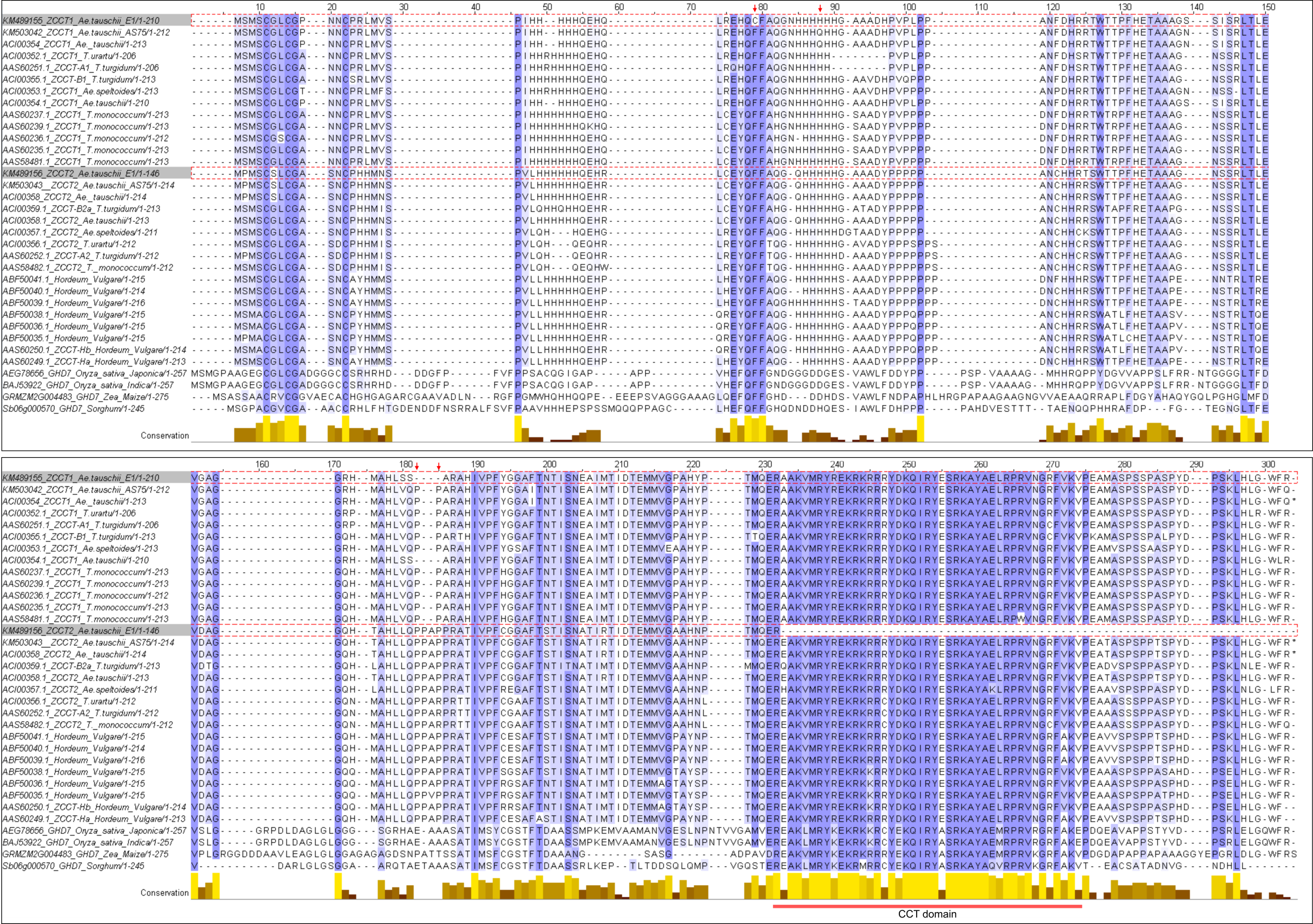
**

**Fig S1. *Mutations in the VRN-D2 locus of Ae. tauschii E1.*** Multiple alignments of *ZCCT1* and *ZCCT2.* Arrows indicate the *ZCCT1* mutations (F36C, H45Q and a 2 bp deletion) in *Ae.tauschii* E1. Numbers in protein sequences names correspond to GenBank identifiers, numbers after slash correspond to length of sequence.

**Fig S2. Multiple sequence alignment of *ZCCT1* genomic sequence in *Ae. tauschii* E1 (KM489155), *Ae. tauschii* AS75 (KM503042) and *Ae. tauschii* AL8/78 (plants.ensembl.org Scaffold12030).** Exons are highlighted in colors (*yellow* and *green*). Start and Stop codons are highlighted in *red*, intron deletion in *Ae. tauschii* E1 is highlighted in *gray* color.

ZCCT1_AL8/78_Scaffold12030 GCTAATTCCCATATTGATTCTTTTTTCTGGCCTGAGAGCTCAGTGCTGCC

ZCCT1_Ae.tauschii_AS75 GCTAATTCCCATATTGATTCTTTTTTCTGGCCTGAGAGCTCAGTGCTGCC

ZCCT1_Ae.tauschii_E1 GCTAATTCCCATATTGATTCTTTTTTCTGGCCTGAGAGCTCAGTGCTGCC

**************************************************

ZCCT1_AL8/78_Scaffold12030 TATATGCAGTGCATGTGAGAGAGGCACAGTACAGCCCTAGCTACTACTAC

ZCCT1_Ae.tauschii_AS75 TATATGCAGTGCATGTGAGAGAGGCACAGTACAGCCCTAGCTACTACTAC

ZCCT1_Ae.tauschii_E1 TATATGCAGTGCATGTGAGAGAGGCACAGTACAGCCCTAGCTACTACTAC

**************************************************

ZCCT1_AL8/78_Scaffold12030 AAGTACCTTGGTAGTTACTGGTACTCATAACTGCCTCTTCTTCTTCCTCG

ZCCT1_Ae.tauschii_AS75 AAGTACCTTGGTAGTTACTGGTACTCATAACTGCCTCTTCTTCTTCCTCG

ZCCT1_Ae.tauschii_E1 AAGTACCTTGGTAGTTACTGGTACTCATAACTGCCTCTTCTTCTTCCTCG

**************************************************

ZCCT1_AL8/78_Scaffold12030 ACATCTCTCCTACTCGGCTCCTCCACGCACCAGACTACACCAGGAAAAAA

ZCCT1_Ae.tauschii_AS75 ACATCTCTCCTACTCGGCTCCTCCACGCACCAGACTACACCAGGAAAAAA

ZCCT1_Ae.tauschii_E1 ACATCTCTCCTACTTGGCTCCTCCACGCACCAGACTACACCAGGAAAAAA

************** ***********************************

ZCCT1_AL8/78_Scaffold12030 CAAACAAGTAAGCAACCCTTGGAGCTAGCTAGCAGTATGTCCATGTCATG

ZCCT1_Ae.tauschii_AS75 CAAACAAGCAAGCAACCCTTGGAGCTAGCTAGCAGTATGTCCATGTCATG

ZCCT1_Ae.tauschii_E1 CAAACAAGCAAGCAACCCTTGGAGCTAGCTAGCAGTATGTCCATGTCATG

******** *****************************************

ZCCT1_AL8/78_Scaffold12030 CGGTTTGTGCGGCCCCAACAACTGCCCGCGCCTCATGGTCTCGCCCATTC

ZCCT1_Ae.tauschii_AS75 CGGTTTGTGCGGCCCCAACAACTGCCCGCGCCTCATGGTCTCGCCCATTC

ZCCT1_Ae.tauschii_E1 CGGTTTGTGCGGCCCCAACAACTGCCCGCGCCTCATGGTCTCGCCCATTC

**************************************************

ZCCT1_AL8/78_Scaffold12030 ATCATCACCATCATCAGGAGCACCAGCTACGCGAGCACCAGTTCTTCGCC

ZCCT1_Ae.tauschii_AS75 ATCATCACCATCATCAGGAGCACCAGCTACGCGAGCACCAGTTCTTCGCC

ZCCT1_Ae.tauschii_E1 ATCATCACCATCATCAGGAGCACCAGCTACGCGAGCACCAGTGCTTCGCC

****************************************** *******

ZCCT1_AL8/78_Scaffold12030 CAAGGCAACCACCACCACCAGCACCATGGCGCGGCAGCAGACCACCCAGT

ZCCT1_Ae.tauschii_AS75 CAAGGCAACCACCACCACCAGCACCATGGCGCGGCAGCAGACCACCCAGT

ZCCT1_Ae.tauschii_E1 CAAGGCAACCACCACCACCACCACCATGGCGCGGCAGCAGACCACCCAGT

******************** *****************************

ZCCT1_AL8/78_Scaffold12030 GCCACTGCCGCCAGCCAACTTCGACCACCGCAGAACATGGACCACACCAT

ZCCT1_Ae.tauschii_AS75 GCCACTGCCGCCAGCCAACTTCGACCACCGCAGAACATGGACCACACCAT

ZCCT1_Ae.tauschii_E1 GCCACTGCCGCCAGCCAACTTCGACCACCGCAGAACATGGACCACACCAT

**************************************************

ZCCT1_AL8/78_Scaffold12030 TTCATGAAACAGCAGCTGCAGGGAGCAGCATCAGCAGGCTCACGCTGGAG

ZCCT1_Ae.tauschii_AS75 TTCATGAAACAGCAGCTGCAGGGAACAGCATCAGCAGGCTCACGCTGGAG

ZCCT1_Ae.tauschii_E1 TTCATGAAACAGCAGCTGCAGGGAGCAGCATCAGCAGGCTCACGCTGGAG

************************ *************************

ZCCT1_AL8/78_Scaffold12030 GTGGGCGCAGGCGGCCGACACATGGCTCACCT------GTCATCGGCAAG

ZCCT1_Ae.tauschii_AS75 GTGGGCGCAGGCGGCCGACACATGGCTCACCTAGTGCAGCCACCGGCAAG

ZCCT1_Ae.tauschii_E1 GTGGGCGCAGGCGGCCGACACATGGCTCACCT------GTCATCGGCAAG

******************************** * ** *******

ZCCT1_AL8/78_Scaffold12030 AGCCCACATCGTAAGTAGTACTAGTACTGCTTAATTGTTTCATCTCTTGC

ZCCT1_Ae.tauschii_AS75 AGCCCACATCGTAAGTAGTACTAGTACTGCTTAATTGTTTCATCTCTTGC

ZCCT1_Ae.tauschii_E1 AGCCCACATCGTAAGTAGTACTAGTACTGCTTAATTGTTTCATCTCTTGC

**************************************************

ZCCT1_AL8/78_Scaffold12030 CGATGGATGCGTCCACGGCTCCCTCCTTAAAATTCCCCACCTAATTAATG

ZCCT1_Ae.tauschii_AS75 CGATGGATGCGTCCACGGCTTCCTCCTTAAAATTCCCCACCTAATTAATG

ZCCT1_Ae.tauschii_E1 CGATGGATGCGTCCACGGCTTCCTCCTTAAAATTCCCCACCTAATTAATG

******************** *****************************

ZCCT1_AL8/78_Scaffold12030 TCTATCTACACCCACTACAAAAAATAGCACCATGTAACCATCTCATATAT

ZCCT1_Ae.tauschii_AS75 TCTATCTGCACCCACTACAAAAAATAGCACCATGTAACCATCTCATATAT

ZCCT1_Ae.tauschii_E1 TCTATCTACACCCACTACAAAAAATAGCACCATGTAACCATCTCATATAT

******* ******************************************

ZCCT1_AL8/78_Scaffold12030 CTGTCACATAATTCTGTTAATTTACGCTGCTCAATTGTTCTTCTGAAAAA

ZCCT1_Ae.tauschii_AS75 CTGTCACATAATTCTGTTAATTTACGCTGCTCAATTGTTCTTCTGAAAAA

ZCCT1_Ae.tauschii_E1 CTGTCACATAATTCTGTTAATTTACGCTGCTCAATTGTTCTTCTGAAAAA

**************************************************

ZCCT1_AL8/78_Scaffold12030 GATATACGGGAATGGATCTTGATATTCTTTAATTCTCTATGGAGCCATAG

ZCCT1_Ae.tauschii_AS75 GATATACGGGAATGGATCTTGATATTCTTTAATTCTCTATGGAGGCATAG

ZCCT1_Ae.tauschii_E1 GATATACGGGAATGGATCTTGATATTCTTTAATTCTCTATGGAGGCATAG

******************************************** *****

ZCCT1_AL8/78_Scaffold12030 AATTTGTGTTTTGTATTACTCCCTCCGTTCGGAATTACTTGTCACGAAAA

ZCCT1_Ae.tauschii_AS75 AATTTGTGTTTTGTATTACTCCCTCCGTTCGGAATTAATTGTCACAGAAA

ZCCT1_Ae.tauschii_E1 AATTTGTGTTTTGTATTACTCCCTCCGTTCGGAATTACTTGTCACGGAAA

************************************* ******* ***

ZCCT1_AL8/78_Scaffold12030 TGGATGTATCTAGACATATTTTAGTTCTAGATACATCCATTTCCGAGACA

ZCCT1_Ae.tauschii_AS75 TGGATGTATCTAGACATATTTTAGTTCTAGATACATCCATTTCCGAGACA

ZCCT1_Ae.tauschii_E1 TGGATGTATCTAGACATATTTTAGTTCTAGATACATCCATTTCCGAGACA

**************************************************

ZCCT1_AL8/78_Scaffold12030 AGTAATTCCGAACGGAGGGAGTAGTTGATAAAGAATTGTATGGGTTGTCA

ZCCT1_Ae.tauschii_AS75 AGTAATTCCGAACGGAGGGAGTAGTTGATAAAGAATTGTATGGGTTGTCA

ZCCT1_Ae.tauschii_E1 AGTAATTCCGAACGGAGGGAGTAGTTGATAAAGAATTGTATGGGTTGTCA

**************************************************

ZCCT1_AL8/78_Scaffold12030 CTCAAATCATCAGTCATACATATATACTTATTTCATTTTATTTGACCAAC

ZCCT1_Ae.tauschii_AS75 CTCAAATCATCAGTCATACATATATACTTATTTCATTTTATTTGACCAAC

ZCCT1_Ae.tauschii_E1 CTCAAATCATCAGTCATACATATATACTTATTTCATTTTATTTGACCAAC

**************************************************

ZCCT1_AL8/78_Scaffold12030 AACAAGGTAATCAGTCATACATGCATAATGAAAATTTGACTTGTGTGCAA

ZCCT1_Ae.tauschii_AS75 AGCAAGGTAATCAGTCATACATGCATAATGAAAATTTGACTTGTGTGCAA

ZCCT1_Ae.tauschii_E1 AACAAGGTAATCAGTCATACATGCATAATGAAAATTTGACTTGTGTGCAA

* ************************************************

ZCCT1_AL8/78_Scaffold12030 TAACTAACCAACTCGACCGGCACAGCTGGGGCAAGACTTTAATCAAGCTG

ZCCT1_Ae.tauschii_AS75 TAACTAACCAACTCGACCGGCACAGCTGGGGCAAGACTTTAATCAAGCTG

ZCCT1_Ae.tauschii_E1 TAACTAACCAACTCGACCGGCACAGCTGGGGTAAGACTTTAATCAAGCTG

******************************* ******************

ZCCT1_AL8/78_Scaffold12030 CTAGCTGGGGCAAGACTTTAATCAAGCTGCTAGCTAGAGCTTAATAATAT

ZCCT1_Ae.tauschii_AS75 CTAGCTGGGGCAAGACTTTAATCAAGCTGCTAGCTAGAGCTTAATAATAT

ZCCT1_Ae.tauschii_E1 CTAGCTGGGGCAAGACTTTAATCAAGCTGCTAGCTAGAGCTTAATAATAT

**************************************************

ZCCT1_AL8/78_Scaffold12030 AACATATCTCTTTATTGGATTAAGCAATACATATGCTCAATTCTCAACTT

ZCCT1_Ae.tauschii_AS75 AACATATCTCTTTATTGGATTAAGCAATACATATGCTCAATTCTCAACTT

ZCCT1_Ae.tauschii_E1 AACATATCTCTTTATTGGATTAAGCAATACATATGCTCAATTCTCAACTT

**************************************************

ZCCT1_AL8/78_Scaffold12030 GTCAGTATCTATCTGGAGTCCACACCTTTATGATAATTAATTGACAAAGT

ZCCT1_Ae.tauschii_AS75 GTCAGTATCTATCTGGAGTCCACACCTTTATGATAATTAATTGACAAAGT

ZCCT1_Ae.tauschii_E1 GTCAGTATCTATCTGGAGTCCACACCTTTATGGTAATTAATTGACAAAGT

******************************** *****************

ZCCT1_AL8/78_Scaffold12030 TTTGTGAAATGGACAATATACATACTGGATCGATGCACCCTCTTTCTCAT

ZCCT1_Ae.tauschii_AS75 TTTGTGAAATGGACAATATACATACTGGATCGATGCACCCTCTTTCTCAT

ZCCT1_Ae.tauschii_E1 TTTGTGAAATGGACAATATACATACTGGATCGATGCACCCTCTTTCTCAT

**************************************************

ZCCT1_AL8/78_Scaffold12030 TTTATGTGGTCATTATGAATTTAATTGTTATTTTGTATTTAAATTTTACC

ZCCT1_Ae.tauschii_AS75 TTTATGTGGTCATTATGAATTTAATTGTTATTTTGTATTTAAATTTTACC

ZCCT1_Ae.tauschii_E1 TTTATGTGGTCATTATGAATTTAATTGTTATTTTGTATTTAAATTTTACC

**************************************************

ZCCT1_AL8/78_Scaffold12030 TTGAGCTAGTTTTGCAAGTCTGTAGCTCATATATAACTGATACTACTCCC

ZCCT1_Ae.tauschii_AS75 TTGAGCTAGTTTTGCAAGTCTGTAGCTCATATATAACTGATACTACTCCC

ZCCT1_Ae.tauschii_E1 TTGAGCTAGTTTTGCAAGTCTGTAGCTCATATATAACTGATACTACTCCC

**************************************************

ZCCT1_AL8/78_Scaffold12030 CACGATAGCTTGCGTAGTGGCCGGGTGATCGATCTACCGAGTTCATAAAA

ZCCT1_Ae.tauschii_AS75 CACGATAGCTTGCGTAGTGGCCGGGTGATCGATCTACCGAGTTCATAAAA

ZCCT1_Ae.tauschii_E1 CACGATAGCTTGTGTAGTGGCCGGGTGATCGATCTACCGAGTTCATAAAA

************ *************************************

ZCCT1_AL8/78_Scaffold12030 CTGATCGAGATCGGGTCCAAAAATGAACAAAACCATACAGAATGGAAAGA

ZCCT1_Ae.tauschii_AS75 CTGATCGAGATCGGGTCCAAAAATGAACAAAACCATACAGAATGGAAAGA

ZCCT1_Ae.tauschii_E1 CTGATCGAGATCGGGTCCAAAAATGAACAAAACCATACAGAATGGAAAGA

**************************************************

ZCCT1_AL8/78_Scaffold12030 AAAGAAGATCCTTGTTTAGTTAGTTTGCATCAGGAAATTGCCTACTTAGT

ZCCT1_Ae.tauschii_AS75 AAAGAAGATCCTTGTTTAGTTAGTTTGCATCAGGAAATTGCCTACTTAGT

ZCCT1_Ae.tauschii_E1 AAAGAAGATCCTTGTTTAGTTAGTTTGCATCAGGAAATTGCCTACTTAGT

**************************************************

ZCCT1_AL8/78_Scaffold12030 TACTCGCTATCAATCTTTTGAACATGGCATGTTCATCCCAAACGGACCCA

ZCCT1_Ae.tauschii_AS75 TACTCGCTATCAATCTTTTGAACATGGCATGTTCATCCCAAACGGACCCA

ZCCT1_Ae.tauschii_E1 TACTCGCTATCAATCTTTTGAACATGGCATGTTCATCCCAAACGGACCCA

**************************************************

ZCCT1_AL8/78_Scaffold12030 GATCACAATTGCTGAGGAAGTTACACCTTTTAAAAACTCATAAAACTGTA

ZCCT1_Ae.tauschii_AS75 GATCACAATTGCTGATGAAGTTACACCTTTTAAAAACTCATAAAACTGTA

ZCCT1_Ae.tauschii_E1 GATCACAATTGCTGATGAAGTTACACCTTTTAAAAACTCATAAAACTGTA

*************** **********************************

ZCCT1_AL8/78_Scaffold12030 CATACATGTACAGGGCTACACGCATGTACATAATACACCTAATTGAAACG

ZCCT1_Ae.tauschii_AS75 CATACATGTACAGGGCTACACGCATGTACATAATACACCTAATTGAAACG

ZCCT1_Ae.tauschii_E1 CATACATGTACAGGGCTACACGCATGTACATAATACACCTAATTGAAACG

**************************************************

ZCCT1_AL8/78_Scaffold12030 TATATTCGTAGACCAATTGATTTTGGACTGTGCACATCTTTGGAAAAAAA

ZCCT1_Ae.tauschii_AS75 TATATTCGTAGACCAATTGATTTTGGACTGTGCGCATCTTTGGAAAAAAA

ZCCT1_Ae.tauschii_E1 TATATTCGTAGACCAATTGATTTTGGACTGTGCGCATCTTTGGAAAAAAA

********************************* ****************

ZCCT1_AL8/78_Scaffold12030 TGCCAGGGGAGTTGTTATCTTCCGCTGTCTAAAAATAGAATAGTTACAAT

ZCCT1_Ae.tauschii_AS75 TGCCAGGGGAGTTGTTATCTTCCGCTGTCTAAAAATAGAATAGTTACAAT

ZCCT1_Ae.tauschii_E1 TGCCAGGGGAGTTGTTATCTTCCGCTGTCTAAAAATAGAATAGTTACAAT

**************************************************

ZCCT1_AL8/78_Scaffold12030 CAAGTGCACCTCTGAATGAAAATGGATCATTTTCTAGTTAATTAGAGACC

ZCCT1_Ae.tauschii_AS75 CAAGTGCACCTCTGAATGAAAATGGATCATTTTCTAGTTAATTAGAGACT

ZCCT1_Ae.tauschii_E1 CAAGTGCACCTCTGAATGAAAATGGATCATTTTCTAGTTAATTAGAGACT

*************************************************

ZCCT1_AL8/78_Scaffold12030 AATTAGATACTTCATAAACAGGGGAATATCAAGTACGTATCTGCTACCCA

ZCCT1_Ae.tauschii_AS75 AATTAGATACTTCATAAACAGGGGAATATCAAGTACGTATCTGCTACCCA

ZCCT1_Ae.tauschii_E1 AATTAAATACTTCATAAACAGGGGAATATCAAGTACGTATCTGCTACCCA

***** ********************************************

ZCCT1_AL8/78_Scaffold12030 TAAGAAAGTACATAACTGCGATCTTATGATTATTTCCCTCTTGATGTTCA

ZCCT1_Ae.tauschii_AS75 TAAGAAAGTACATAACTGCGATCTTATGATTATTTCCCTCTTGATGTTCA

ZCCT1_Ae.tauschii_E1 TAAGAAAGTACATAACTGC------------------------ATGTTCA

******************* *******

ZCCT1_AL8/78_Scaffold12030 GGTGCCATTTTACGGAGGTGCATTCACAAACACTATTAGCAATGAAGCAA

ZCCT1_Ae.tauschii_AS75 GGTGCCATTTTACGGAGGTGCAATCACAAACACTATTAGCAATGAAGCAA

ZCCT1_Ae.tauschii_E1 GGTGCCATTTTACGGAGGTGCATTCACAAACACTATTAGCAATGAAGCAA

********************** ***************************

ZCCT1_AL8/78_Scaffold12030 TCATGACTATTGACACAGAGATGATGGTGGGGCCTGCCCATTATCCCACA

ZCCT1_Ae.tauschii_AS75 TCATGACTATTGACACAGAGATGATGGTGGGGCCTGCCCATTATCCCACA

ZCCT1_Ae.tauschii_E1 TCATGACTATTGACACAGAGATGATGGTGGGGCCTGCCCATTATCCCACA

**************************************************

ZCCT1_AL8/78_Scaffold12030 ATGCAGGAGAGAGCGGCGAAGGTGATGAGGTATAGGGAGAAGAGGAAGAG

ZCCT1_Ae.tauschii_AS75 ATGCAGGAGAGAGCGGCAAAGGTGATGAGGTATAGGGAGAAGAGGAAGAG

ZCCT1_Ae.tauschii_E1 ATGCAGGAGAGAGCGGCAAAGGTGATGAGGTATAGGGAGAAGAGGAAGAG

***************** ********************************

ZCCT1_AL8/78_Scaffold12030 GCGGCGCTATGACAAGCAAATCCGATACGAGTCCAGAAAAGCTTACGCTG

ZCCT1_Ae.tauschii_AS75 GCGGCGCTATGACAAGCAAATCCGATACGAGTCCAGAAAAGCTTACGCTG

ZCCT1_Ae.tauschii_E1 GCGGCGCTATGACAAGCAAATCCGATACGAGTCCAGAAAAGCTTACGCCG

************************************************ *

ZCCT1_AL8/78_Scaffold12030 AGCTCCGACCACGGGTCAACGGTCGCTTCGTCAAGGTACCCGAAGCCATG

ZCCT1_Ae.tauschii_AS75 AGCTCCGGCCACGGGTCAACGGCCGCTTCGTCAAGGTACCCGAAGCCATG

ZCCT1_Ae.tauschii_E1 AGCTCCGGCCACGGGTCAACGGCCGCTTCGTCAAGGTACCCGAAGCCATG

******* ************** ***************************

ZCCT1_AL8/78_Scaffold12030 GCATCGCCATCATCTCCAGCTTCGCCCTATGATCCTAGTAAACTTCATCT

ZCCT1_Ae.tauschii_AS75 GCATCGCCATCATCTCCAGCTTCGCCCTATGATCCTAGTAAACTTCATCT

ZCCT1_Ae.tauschii_E1 GCATCGCCATCATCTCCAGCTTCGCCCTATGATCCTAGTAAACTTCATCT

**************************************************

ZCCT1_AL8/78_Scaffold12030 TGGATGGCTCCGGTAATTTATAGCACAAGCCAGATAAAATGATAACATAT

ZCCT1_Ae.tauschii_AS75 TGGATGGTTCCAGTAATTTATAGCACAAGCCAGATAAAATGATAACATAT

ZCCT1_Ae.tauschii_E1 TGGATGGTTCCGGTAATTTATAGCACAAGCCAGATAAAATGATAACATAT

******* *** **************************************

ZCCT1_AL8/78_Scaffold12030 TTACTTTTGATTGATCCACCTGTGAAGCAGTTGTTCATCAAAGTAAAATA

ZCCT1_Ae.tauschii_AS75 TTACTTTTGATTGATCCACCTGTGAAGCAGTTGTTCATCAAAGTAAAATA

ZCCT1_Ae.tauschii_E1 TTACTTTTGATTGATCCACCTGTGAAGCAGTTGTTCATCAAAGTAAAATA

**************************************************

ZCCT1_AL8/78_Scaffold12030 AGTCGGTTAGTGGTTGATCGATTGGAGCCATTATGTTGATTTGACTATTT

ZCCT1_Ae.tauschii_AS75 AGTCGGTTAGT---------------------------------------

ZCCT1_Ae.tauschii_E1 AGTCGGTTAGT---------------------------------------

***********

**Table S1. Primer sequences and PCR conditions for markers, cDNAs and genomic sequencing of *ZCCT-1* and *ZCCT-2* from *Ae. tauschii.***

| **Gene name** | **Target** | | **Primer name** | **Primer sequence (5' to 3')** | **Ann. T. (⁰C)** | **Ext. time (s)** |
| --- | --- | --- | --- | --- | --- | --- |
| ***Molecular markers T. aestivum*** | | | |  |  |  |
| *SNF-B2* | Genomic | SNF-B2-3p-F1 | | GTTGGTTCTTACGCTAGCTCT | 62 | 90 |
|  |  | SNF-B2-3p-R2 | | AACCCAATATGTCTTGCTATGA |  |  |
| *ZCCT-D1* | Genomic | ZCCT-D1-E1-CAPS-F1 | | CTTCTTCCTCGACATCTCTCCTA | 62 | 60 |
|  |  | ZCCT-D1-E1-CAPS-R1 | | GGAGGGAGTAATACAAAACACAAAT |  |  |
| ***RNA splicing variants in Ae. tauschii*** | | | |  |  |  |
| *ZCCT-D1* | cDNA | ZCCT-D1-F | | TCCATGTCATGCGGTTTG | 60 | 60 |
|  |  | ZCCT-D1-R | | ACCGGAACCATCCAAGAT |  |  |
| *ZCCT-D2* | cDNA | ZCCT-D2-F | | ATGCCCATGTCATGCAGT | 60 | 60 |
|  |  | ZCCT-D2-R | | TACCGGAACCATCCGAGG |  |  |
| ***Sequencing VRN-D2* from *Ae. tauschii*** | | | |  |  |  |
| *ZCCT-D1* | Exon1 | ZCCT-5P-CF2 | | TATCTATTCCGACGCACTCATTAG | 62 | 90 |
|  |  | ZCCT-R2 | | CTCTCCTGCATTGTGGGATA |  |  |
| *ZCCT-D1* | Exon 2 | ZCCT1-F2 | | CCAGCCAACTTCGACCACCGCAGAA | 62 | 90 |
|  |  | ZCCT-3P-CR3 | | TCTGCTGACCATTTTAAATAGTC |  |  |
| *ZCCT-D1* | Exon 2 | ZCCT1-F3 | | ATCAGCAGGCTCACGCTGGAGGTGGG | 60 | 90 |
|  |  | ZCCT-3P-CR1 | | CACTAACCGACTTATTTTACTTTG |  |  |
| *ZCCT-D2* | Exon 1 | ZCCT1-5P-CF5 | | CTCCACGCACCAAACCACACCAG | 64 | 90 |
|  |  | ZCCT-2-R2 | | CTCTCCTGCATCGTCGGATT |  |  |
| *ZCCT-D2* | Exon 2 | ZCCT2-F1 | | CCACCATCATCAGGAACACCG | 62 | 90 |
|  |  | ZCCT-3P-CR4 | | TCTGCTGACCTTTTGAAATAGTC |  |  |
| *ZCCT-D2* | Exon 2 | ZCCT2-F2 | | CCGGCCAACTGCCACCACCGCAGAT | 64 | 90 |
|  |  | ZCCT-3P-CR4 | | TCTGCTGACCTTTTGAAATAGTC |  |  |
| ***qRT-PCR*** |  |  | |  |  |  |
| *ZCCT-B2* |  | qZCCT-B2-F2 | | ACCCTGGAGGTGGACGCT |  |  |
|  |  | qZCCT-BD2-R2 | | GAATGGCACGATGGTGGC |  |  |
| *ZCCT-D2* |  | qZCCT-D2-F1 | | CGCGGGCGGACAACACAC |  |  |
|  |  | qZCCT-BD2-R1 | | GAATGGCACGATGGTGGC |  |  |

*Touch-down protocol for PCR includes an additional 94 ºC for 5 m, 12 cycles of initial touch-down, with a reduction of 0.5 ºC per cycle (6 ºC total from final annealing T), then followed by 40 cycles of 94 ºC for 5 m, annealing temperature for 30 s and a final extension time of 7 m at 72 ºC.
